# Supplementary material for: The characteristic of patulous eustachian tube patients diagnosed by the JOS diagnostic criteria
Source: PLoS One. 2019 Dec 27;14(12):e0226908. doi: 10.1371/journal.pone.0226908 (PMC6934284; doi:10.1371/journal.pone.0226908)
Supplement: S1 Table — (DOCX) [file pone.0226908.s001.docx]

S1 Table. Summary of the clinical features of patients (raw data)

1: positive, 0: negative

| **Case** | **sex** | **age** | **R/L** | **initial or second** | **autophony** | **aural fullness** | **respiration** | **PHI 10** | **otoscope** | **positon change** | **obstraction** | **TM movement** | **sono** | **sono platau** | **Ohta10dB** | **TTAG** | | **CT** | |
| --- | --- | --- | --- | --- | --- | --- | --- | --- | --- | --- | --- | --- | --- | --- | --- | --- | --- | --- | --- |
| 1 | 1 | 63 | 1 | 0 | 1 | 1 | 0 |  |  | 1 |  | 1 | 0 | 0 | 0 | 0 | | 0 | |
| 2 | 0 | 34 | 0 | 0 | 0 | 1 | 0 | 40 |  | 1 |  | 0 | 0 | 0 |  | 1 | | 0 | |
| 3 | 0 | 34 | 1 | 0 | 1 | 1 | 0 | 40 |  | 1 |  | 0 | 0 | 0 |  | 1 | | 0 | |
| 4 | 0 | 69 | 1 | 1 | 1 | 1 | 0 | 8 | 1 | 1 |  | 0 | 0 | 0 | 1 | 1 | |  | |
| 5 | 1 | 78 | 0 | 0 | 1 | 0 | 1 | 2 |  | 1 |  |  | 0 | 1 | 0 | 0 | | 1 | |
| 6 | 1 | 78 | 1 | 0 | 1 | 0 | 1 | 2 |  | 1 |  | 1 | 0 | 0 | 0 | 0 | | 1 | |
| 7 | 1 | 66 | 1 | 0 | 1 | 1 | 0 | 30 | 1 | 1 |  | 1 | 1 | 0 |  | 0 | | 1 | |
| 8 | 1 | 66 | 0 | 0 | 1 | 0 | 0 | 20 |  | 1 |  | 1 | 1 | 0 |  | 1 | | 1 | |
| 9 | 0 | 62 | 1 | 0 | 1 | 1 | 0 | 18 | 0 | 1 |  | 1 | 1 | 0 | 0 | 1 | |  | |
| 10 | 0 | 43 | 0 | 0 | 1 | 1 | 1 |  |  | 1 |  | 0 | 0 | 0 |  | 1 | | 1 | |
| 11 | 1 | 23 | 1 | 1 | 1 | 1 | 1 | 28 | 1 | 1 |  | 0 | 0 | 0 | 0 | 1 | |  | |
| 12 | 0 | 45 | 0 | 0 | 1 | 1 | 1 | 36 | 0 | 1 |  | 1 | 0 | 1 |  | 0 | | 1 | |
| 13 | 0 | 45 | 1 | 0 | 1 | 1 | 1 | 36 |  | 1 |  | 1 | 0 | 1 |  | 1 | | 0 | |
| 14 | 1 | 41 | 0 | 0 | 1 | 1 | 1 | 20 |  | 1 |  | 1 | 1 | 0 |  | 1 | | 1 | |
| 15 | 1 | 38 | 0 | 0 | 1 | 1 |  | 40 | 1 | 1 |  | 1 | 0 | 0 |  | 1 | | 1 | |
| 16 | 1 | 38 | 1 | 0 | 1 | 1 |  | 40 |  | 1 |  |  | 1 | 0 |  | 1 | | 1 | |
| 17 | 1 | 49 | 0 | 0 | 1 | 1 | 1 | 34 |  | 1 |  | 1 | 0 | 0 |  | 1 | | 1 | |
| 18 | 1 | 49 | 1 | 0 | 1 | 1 | 1 | 10 |  | 1 |  | 1 | 0 | 0 |  | 1 | | 1 | |
| 19 | 0 | 21 | 0 | 0 | 1 | 1 | 1 | 30 |  | 1 |  | 1 | 1 | 0 | 0 | 1 | | 1 | |
| 20 | 0 | 33 | 1 | 0 | 1 | 1 | 1 | 14 |  | 1 |  | 1 | 0 | 0 |  | 0 | | 0 | |
| 21 | 1 | 27 | 1 | 0 | 1 | 1 | 1 | 32 |  | 1 |  | 0 | 0 | 0 |  | 1 | 1 | |  |
| 22 | 0 | 72 | 0 | 0 | 1 | 1 | 1 | 8 | 1 | 1 |  | 1 | 0 | 0 |  | 1 | 1 | |  |
| 23 | 0 | 72 | 1 | 0 | 0 | 1 | 1 | 28 | 1 | 1 |  | 1 | 1 | 0 |  | 1 | 1 | |  |
| 24 | 1 | 39 | 1 | 0 | 1 | 1 | 1 | 20 |  | 1 |  | 0 | 0 | 1 | 0 | 1 | 0 | |  |
| 25 | 1 | 19 | 0 | 0 | 1 | 1 | 1 | 24 |  | 1 |  | 1 | 1 | 0 | 1 | 1 | 1 | |  |
| 26 | 1 | 19 | 1 | 1 | 1 | 1 | 0 | 22 |  | 1 |  | 0 | 1 | 1 | 0 | 1 |  | |  |
| 27 | 1 | 70 | 0 | 0 | 1 | 1 | 1 | 10 |  | 1 |  | 1 | 1 | 0 | 1 | 1 | 1 | |  |
| 28 | 1 | 70 | 1 | 0 | 1 | 1 | 1 | 20 |  | 1 |  | 1 | 1 | 0 | 1 | 1 | 1 | |  |
| 29 | 1 | 12 | 0 | 0 | 1 | 1 | 1 | 28 |  | 0 | 1 |  | 1 | 0 | 0 | 1 | 1 | |  |
| 30 | 1 | 12 | 1 | 0 | 1 | 1 | 1 | 12 |  | 0 | 1 |  | 1 | 1 | 0 | 1 | 1 | |  |
| 31 | 1 | 38 | 1 | 1 | 1 | 1 | 1 | 6 |  | 1 |  | 1 | 0 | 0 | 0 | 0 |  | |  |
| 32 | 0 | 62 | 0 | 0 | 1 | 1 | 1 | 36 |  | 1 |  | 0 | 1 | 0 | 1 | 1 | 1 | |  |
| 33 | 0 | 62 | 1 | 0 | 1 | 1 | 1 | 32 |  | 1 |  | 1 | 1 | 0 | 1 | 1 | 1 | |  |
| 34 | 1 | 35 | 1 | 0 | 1 | 1 | 1 | 16 |  | 1 |  | 1 | 1 | 0 | 0 | 1 | 1 | |  |
| 35 | 1 | 41 | 0 | 1 | 1 | 0 | 0 | 10 | 0 | 1 |  | 1 | 1 | 0 | 0 | 0 |  | |  |
| 36 | 1 | 41 | 1 | 0 | 1 | 1 | 1 | 32 | 1 | 1 |  | 0 | 1 | 0 | 0 | 1 | 1 | |  |
| 37 | 0 | 69 | 0 | 0 | 1 | 1 | 1 | 28 |  | 1 |  | 1 | 1 | 0 | 1 | 1 | 1 | |  |
| 38 | 0 | 58 | 0 | 1 | 1 | 1 | 1 | 26 |  | 1 |  | 1 | 0 | 1 | 0 | 1 |  | |  |
| 39 | 0 | 58 | 1 | 0 | 1 | 1 | 1 | 34 | 0 | 1 |  | 1 | 1 | 0 | 1 | 1 | 1 | |  |
| 40 | 1 | 38 | 0 | 0 | 1 | 0 | 1 | 40 | 0 | 1 |  | 0 | 1 | 0 | 1 | 1 | 1 | |  |
| 41 | 1 | 38 | 1 | 0 | 1 | 0 | 1 | 28 | 0 | 1 |  | 0 | 1 | 0 | 1 | 1 | 1 | |  |
| 42 | 0 | 52 | 0 | 0 | 1 | 1 | 1 | 28 | 1 | 1 |  |  | 0 | 0 | 0 | 1 | 0 | |  |
| 43 | 0 | 79 | 0 | 0 | 1 | 1 | 1 | 0 |  | 1 |  |  | 0 | 1 | 0 | 1 | 1 | |  |
| 44 | 0 | 79 | 1 | 0 | 1 | 1 | 1 | 34 |  | 1 |  |  | 1 | 0 | 0 | 1 | 1 | |  |
| 45 | 0 | 54 | 0 | 0 | 1 | 1 | 1 |  |  | 1 |  | 0 | 1 | 0 | 1 | 1 | 1 | |  |
| 46 | 0 | 53 | 1 | 0 | 1 | 1 | 0 | 26 |  | 1 |  | 1 | 0 | 0 | 0 | 0 | 0 | |  |
| 47 | 1 | 70 | 0 | 0 | 1 | 1 | 1 | 22 | 0 | 0 | 1 |  | 0 | 1 | 1 | 0 | 0 | |  |
| 48 | 1 | 70 | 1 | 0 | 1 | 1 | 0 | 38 |  | 0 | 1 | 0 | 1 | 1 | 0 | 0 | 1 | |  |
| 49 | 1 | 41 | 1 | 0 | 0 |  | 1 | 12 |  | 1 |  | 1 | 0 | 0 | 1 | 1 | 1 | |  |
| 50 | 1 | 45 | 0 | 0 | 1 | 1 | 1 | 34 | 1 | 1 |  |  | 0 | 0 | 1 | 1 | 0 | |  |
| 51 | 1 | 64 | 1 | 0 | 1 | 1 | 1 | 38 |  | 1 |  | 0 | 0 | 0 | 0 | 1 | 0 | |  |
| 52 | 1 | 25 | 0 | 0 | 1 | 1 | 1 | 40 |  | 1 |  | 1 | 0 | 0 | 1 | 1 | 0 | |  |
| 53 | 1 | 25 | 1 | 0 | 1 | 1 | 1 | 40 |  | 1 |  | 1 | 0 | 0 | 1 | 1 | 1 | |  |
| 54 | 1 | 23 | 0 | 0 | 1 | 1 | 1 | 40 |  | 1 |  | 0 | 0 | 0 | 1 | 1 | 1 | |  |
| 55 | 1 | 33 | 1 | 0 | 0 | 1 | 0 | 18 |  | 1 |  | 1 | 0 | 0 | 0 | 0 | 0 | |  |
| 56 | 1 | 25 | 0 | 0 | 1 | 1 | 1 | 40 |  | 1 |  |  | 0 | 0 | 0 | 1 | 0 | |  |
| 57 | 0 | 83 | 0 | 0 | 1 | 1 | 1 | 30 | 0 | 1 |  | 1 | 0 | 0 | 1 | 1 | 1 | |  |
| 58 | 1 | 88 | 0 | 0 | 1 | 1 | 1 | 40 |  | 1 |  | 1 | 0 | 0 | 0 | 0 | 1 | |  |
| 59 | 1 | 88 | 1 | 0 | 1 | 1 | 1 | 40 |  | 1 |  | 1 | 0 | 0 | 0 | 0 | 1 | |  |
| 60 | 1 | 34 | 1 | 0 | 1 | 1 | 1 | 32 |  | 1 |  | 1 | 0 | 0 | 0 | 0 | 0 | |  |
| 61 | 0 | 64 | 0 | 0 | 1 | 1 | 1 | 8 |  | 0 | 1 | 1 | 0 | 0 | 1 | 1 | 0 | |  |
| 62 | 0 | 54 | 0 | 1 | 1 | 0 | 1 |  |  | 1 |  | 1 | 1 | 0 | 0 | 1 |  | |  |
| 63 | 0 | 54 | 1 | 0 | 1 | 1 | 1 | 38 |  | 1 |  | 1 | 1 | 0 | 0 | 1 | 1 | |  |
| 64 | 1 | 39 | 1 | 0 | 1 | 1 | 1 | 22 |  | 1 |  | 1 | 1 | 0 | 1 | 0 | 1 | |  |
| 65 | 1 | 41 | 1 | 0 | 1 | 1 | 1 | 32 |  | 1 |  | 1 | 1 | 0 | 0 | 0 | 1 | |  |
| 66 | 1 | 74 | 1 | 0 | 0 | 1 | 0 | 4 |  | 1 |  | 0 | 0 | 0 | 1 | 1 | 0 | |  |
| 67 | 0 | 62 | 0 | 0 | 1 | 0 | 0 | 22 | 1 | 1 |  | 0 | 0 | 0 | 0 | 1 | 0 | |  |
| 68 | 0 | 62 | 1 | 0 | 1 | 0 | 1 | 6 | 1 | 1 |  | 0 | 1 | 0 | 1 | 1 | 1 | |  |
| 69 | 0 | 62 | 0 | 0 | 1 | 1 | 1 | 32 |  | 1 |  | 1 | 1 | 0 | 1 | 1 | 1 | |  |
| 70 | 0 | 62 | 1 | 0 | 1 | 1 | 1 | 32 |  | 1 |  | 1 | 1 | 0 | 1 | 1 | 1 | |  |
| 71 | 0 | 39 | 1 | 0 | 1 | 1 | 0 | 38 |  | 1 |  | 0 | 0 | 0 | 0 | 1 | 0 | |  |
| 72 | 1 | 47 | 0 | 0 | 1 | 1 | 1 | 32 | 1 | 1 |  | 0 | 0 | 1 | 0 | 1 | 0 | |  |
| 73 | 1 | 83 | 1 | 0 | 1 | 1 | 1 | 40 |  | 1 |  | 1 | 0 | 0 | 1 | 1 | 1 | |  |
| 74 | 1 | 19 | 0 | 0 | 1 | 1 | 1 | 20 |  | 0 | 1 | 1 | 0 | 0 | 0 | 1 | 0 | |  |
| 75 | 1 | 19 | 1 | 0 | 1 | 1 | 1 | 20 |  | 0 | 1 | 1 | 0 | 0 | 0 | 1 | 0 | |  |
| 76 | 1 | 69 | 0 | 0 | 1 | 1 | 1 | 24 |  | 1 |  | 1 | 1 | 0 | 1 | 1 | 1 | |  |
| 77 | 1 | 65 | 1 | 0 | 1 | 1 | 1 | 34 | 0 | 1 |  | 1 | 1 | 0 | 1 | 1 | 1 | |  |
| 78 | 1 | 30 | 0 | 0 | 1 | 1 | 1 | 14 | 0 | 1 |  | 1 | 1 | 0 | 1 | 0 | 1 | |  |
