# Supplementary material for: The characteristic of patulous eustachian tube patients diagnosed by the JOS diagnostic criteria
Source: PLoS One. 2019 Dec 27;14(12):e0226908. doi: 10.1371/journal.pone.0226908 (PMC6934284; doi:10.1371/journal.pone.0226908)
Supplement: S3 Table — (DOCX) [file pone.0226908.s003.docx]

S3 Table. Summary of results from TTAG, sonotubometry, Ohta method and PHI-10.

| **Case** | **PHI 10** | **sono dB** | **Ohta method** | **EAC/nasal** |
| --- | --- | --- | --- | --- |
| 1 |  | 122 | 0 |  |
| 2 | 40 | 116 |  | 6.06 |
| 3 | 40 | 118 |  | 4.17 |
| 4 | 8 | 106 | 10 | 39.29 |
| 5 | 2 | 108 | 0 |  |
| 6 | 2 | 110 | 7 |  |
| 7 | 30 | 77 |  |  |
| 8 | 20 | 86 |  | 1.73 |
| 9 | 18 | 97 | 3 | 12.07 |
| 10 |  | 102 |  | 8.33 |
| 11 | 28 | 113 | 1 | 13.54 |
| 12 | 36 | 108 |  |  |
| 13 | 36 | 108 |  | 27.56 |
| 14 | 20 | 99 |  | 40.79 |
| 15 | 40 | 100 |  | 31.14 |
| 16 | 40 | 85 |  | 23.96 |
| 17 | 34 | 102 |  | 35.83 |
| 18 | 10 | 103 |  | 34.90 |
| 19 | 30 | 91 | 4 | 31.67 |
| 20 | 14 | 118 |  |  |
| 21 | 32 | 117 |  | 14.88 |
| 22 | 8 | 100 |  | 34.21 |
| 23 | 28 | 98 |  | 3.95 |
| 24 | 20 | 117 | 4 | 18.72 |
| 25 | 24 | 94 | 16 | 35.42 |
| 26 | 22 | 99 | 9 | 40.97 |
| 27 | 10 | 94 | 18 | 27.38 |
| 28 | 20 | 89 | 27 | 19.17 |
| 29 | 28 | 89 | 8 | 41.67 |
| 30 | 12 | 94 | 6 | 51.85 |
| 31 | 6 | 112 | 9 |  |
| 32 | 36 | 87 | 25 | 13.38 |
| 33 | 32 | 98 | 24 | 8.11 |
| 34 | 16 | 97 | 2 | 36.27 |
| 35 | 10 | 98 | 8 |  |
| 36 | 32 | 86 | 1 | 46.88 |
| 37 | 28 | 91 | 21 | 65.00 |
| 38 | 26 | 101 | 9 | 10.83 |
| 39 | 34 | 94 | 12 | 17.08 |
| 40 | 40 | 87 | 15 | 16.21 |
| 41 | 28 | 84 | 15 | 8.83 |
| 42 | 28 | 119 | 0 | 9.14 |
| 43 | 0 | 123 | 0 | 8.90 |
| 44 | 34 | 93 | 3 | 12.50 |
| 45 |  | 84 | 10 | 9.83 |
| 46 | 26 | 122 | 1 |  |
| 47 | 22 | 104 | 18 |  |
| 48 | 38 | 96 | 0 |  |
| 49 | 12 | 104 | 13 | 31.00 |
| 50 | 34 | 113 | 10 | 17.33 |
| 51 | 38 | 109 | 4 | 4.76 |
| 52 | 40 | 101 | 12 | 7.84 |
| 53 | 40 | 104 | 11 | 8.13 |
| 54 | 40 | 111 | 35 | 7.55 |
| 55 | 18 | 113 | 5 |  |
| 56 | 40 | 116 | 3 | 5.00 |
| 57 | 30 | 106 | 21 | 36.81 |
| 58 | 40 | 109 | 6 |  |
| 59 | 40 | 106 | 4 |  |
| 60 | 32 | 109 | 9 |  |
| 61 | 8 | 101 | 12 | 29.17 |
| 62 |  | 94 | 4 | 36.46 |
| 63 | 38 | 89 | 5 | 26.04 |
| 64 | 22 | 97 | 11 |  |
| 65 | 32 | 94 | 6 |  |
| 66 | 4 | 118 | 15 | 2.22 |
| 67 | 22 | 118 | 0 | 13.36 |
| 68 | 6 | 99 | 10 | 20.00 |
| 69 | 32 | 99 | 23 | 31.11 |
| 70 | 32 | 95 | 28 | 25.00 |
| 71 | 38 | 120 | 5 | 23.12 |
| 72 | 32 | 116 | 4 | 6.79 |
| 73 | 40 | 104 | 14 | 9.52 |
| 74 | 20 | 109 | 5 | 41.20 |
| 75 | 20 | 110 | 6 | 30.21 |
| 76 | 24 | 78 | 21 | 8.56 |
| 77 | 34 | 91 | 29 | 17.31 |
| 78 | 14 | 92 | 21 |  |
