# Supplementary material for: The characteristic of patulous eustachian tube patients diagnosed by the JOS diagnostic criteria
Source: PLoS One. 2019 Dec 27;14(12):e0226908. doi: 10.1371/journal.pone.0226908 (PMC6934284; doi:10.1371/journal.pone.0226908)
Supplement: Attachment — Submitted filename: Response to Reviewers ver3.docx [file pone.0226908.s004.docx]

**Response to Reviewers**

**Reviewer #1**

We wish to express our deep appreciation to the reviewer.

I believe figures 5,6,8,9 can be all combined into one with 4 subsections

**Response:**

We have modified as your suggestion.

Figure 2 and 3 can also be combined with subsections

**Response:**

We have modified as your suggestion.

Did your study have any inclusion/exclusion criteria?

**Response:** All patient diagnosed by JOS criteria were included. There was not exclusion criteria.

Abstract page 3

Line 13 – define TTAG

**Response:** We have defined as follows “Tubo-Tympano-Aerodynamic Graphy (TTAG)”.

Line 17 . In ninety-one percent of the ears… -> In 91% of the ears…

**Response:** We have changed “In ninety-one percent of the ears” to “In 91% of the ears”.

Line 18 ….lying / lordotic position… -> do you mean supine? (lordotic is not often used within English literature, please correct to supine within the manuscript)

**Response:** “Lordotic” means bending forward.

We have modified in the text.

Methods page 8

Line 14 Criteria 3C use -> Criteria 3C used

**Response:** We have corrected as your suggestion.

Page 9 Changes in probe tone SPL exceeding 10dB was regarded as

a positive finding of PET – was this something that you defined as significant or previously defined and accepted as a significant finding,

**Response:** There have been no report to define and accepted report as a significant findings. Our results could contribute to define the significant findings.

Line 16 define CT

**Response:** We have defined “CT” as “cone-beam computed tomography (CBCT)”.

Results

Page 12

Line 10 As the three ears exhibited both positive findings… what is meant by “three ears”

**Response:** “Three ears” mean that three ears were positive for both probe tone testing and an open plateau pattern.

We have corrected in the text.

Page 13

It is a very interesting finding that 43 (58.1%) had severe handicap

We wish to express our deep appreciation to the reviewer.

Page 15

Line 13 please change annoying to bothersome

**Response:** We have corrected as your suggestion.

Page 16

Line 14 nostril to EACl - nostril to EAC

**Response:** We have corrected as your suggestion.

References

Please remove number 5 as it is repeated twice

**Response:** We have deleted as your suggestion.

Figure 4

Closed short is not very definite imaging, maybe adding a bracket to show where the region of closed portion of ET is being evaluated

**Response:** We have modified in the figure.

**Reviewer #2**

We wish to express our deep appreciation to the reviewer.

Page 8, Line 4: How was 2B conductive specifically?

**Response:** For example, swab is placed or a small amount of gel is injected into pharyngeal orifice through nasal cavity. Improvement of aural symptoms is regarded as positive.

Page 8, Line 5,6: Presume that any movement of the TM is defined as positive? Please clarify.

**Response:** We have modified as your suggestion.

Page 9, Line 9,10: Please clarify in the text what is being monitored at the level of the EAC (sound transmitted from the nasopharynx?)

**Response:** We have added the sentence “Pressure changes in the external auditory canal (EAC) and the nasopharynx were simultaneously recorded using the manometry mode of the TTAG.”

Page 12, Line 10-12: Three ears were positive for both probe tone testing and an open plateau pattern?

**Response:** “three ears exhibited both positive findings” means “three ears were positive for both probe tone testing and an open plateau pattern.”

We have added the sentence “As three ears were positive for both probe tone testing and an open plateau pattern,”.

Page 14, line 10-13: Any correlation between these delayed “Definite PET” and severity of symptoms? Perhaps less because they were intermittent?

**Response:** PHI-10 score of initial“Definite PET” and delayed “Definite PET” are 26.6±11.5 and 16.7±9.77 (p = 0.054), respectively. Delayed type tended to less severity but not significant because of small number of delayed type.

Page 14, line 13: Consider, “PET symptoms ARE intermittent”.

**Response:** We have corrected as your suggestion.

Page 14, line 17: Consider, “out institute received.

**Response:** We have corrected as your suggestion.

Page 15, line 3: Consider a more formal term in lieu of “Annoying” when referring to PET symptoms.

**Response:** We have changed “annoying” to “bothersome” as your suggestion.

Page 15, line 2-11: Largely restating results. Is there any significance or specific characteristics to the patients that did not improve with postural changes that clinicians should be monitoring for?

**Response:** Thank you very much for useful comment. We also are interested in this point. There are only 4 cases in this study. Further study is needed to elucidate this issue.

Page 16, line 14: EAC1?

**Response:** We have corrected as your suggestion.
